# Prevalence of healthcare-associated infections, estimated incidence and composite antimicrobial resistance index in acute care hospitals and long-term care facilities: results from two European point prevalence surveys, 2016 to 2017

**DOI:** 10.2807/1560-7917.ES.2018.23.46.1800516

**Published:** 2018-11-15

**Authors:** Carl Suetens, Katrien Latour, Tommi Kärki, Enrico Ricchizzi, Pete Kinross, Maria Luisa Moro, Béatrice Jans, Susan Hopkins, Sonja Hansen, Outi Lyytikäinen, Jacqui Reilly, Aleksander Deptula, Walter Zingg, Diamantis Plachouras, Dominique L Monnet, the Healthcare-Associated Infections Prevalence Study Group

**Affiliations:** 1 European Centre for Disease Prevention and Control, Solna, Sweden; 2 Sciensano, Brussels, Belgium; 3 Agenzia sanitaria e sociale regionale – Regione Emilia Romagna, Bologna, Italy; 4 Public Health England, London, United Kingdom; 5 Institute of Hygiene and Environmental Medicine, Charité – University Medicine Berlin, Berlin, Germany; 6 National Institute for Health and Welfare, Department of Health Security, Helsinki, Finland; 7 National Services Scotland, Health Protection Scotland, Glasgow, United Kingdom; 8 Glasgow Caledonian University, Glasgow, United Kingdom; 9 Department of Propaedeutics of Medicine, Nicolaus Copernicus University, Toruń; Ludwik Rydygier Collegium Medicum; Bydgoszcz, Poland; 10 Imperial College London, London, United Kingdom; 11 Members of the Healthcare-Associated Infections Prevalence Study Group are listed at the end of this article

**Keywords:** healthcare-associated infections, HAI, point prevalence survey, PPS, hospitals, long-term care facilities, LTCF, burden, antimicrobial resistance, AMR

## Abstract

Point prevalence surveys of healthcare-associated infections (HAI) and antimicrobial use in the European Union and European Economic Area (EU/EEA) from 2016 to 2017 included 310,755 patients from 1,209 acute care hospitals (ACH) in 28 countries and 117,138 residents from 2,221 long-term care facilities (LTCF) in 23 countries. After national validation, we estimated that 6.5% (cumulative 95% confidence interval (cCI): 5.4–7.8%) patients in ACH and 3.9% (95% cCI: 2.4–6.0%) residents in LTCF had at least one HAI (country-weighted prevalence). On any given day, 98,166 patients (95% cCI: 81,022–117,484) in ACH and 129,940 (95% cCI: 79,570–197,625) residents in LTCF had an HAI. HAI episodes per year were estimated at 8.9 million (95% cCI: 4.6–15.6 million), including 4.5 million (95% cCI: 2.6–7.6 million) in ACH and 4.4 million (95% cCI: 2.0–8.0 million) in LTCF; 3.8 million (95% cCI: 3.1–4.5 million) patients acquired an HAI each year in ACH. Antimicrobial resistance (AMR) to selected AMR markers was 31.6% in ACH and 28.0% in LTCF. Our study confirmed a high annual number of HAI in healthcare facilities in the EU/EEA and indicated that AMR in HAI in LTCF may have reached the same level as in ACH.

## Introduction

In 2016, the European Centre for Disease Prevention and Control (ECDC) estimated that the burden of six main types of healthcare-associated infection (healthcare-associated pneumonia, urinary tract infection, surgical site infection, *Clostridium difficile* infection, neonatal sepsis and primary bloodstream infection)) expressed in disability-adjusted life years (DALYs) in the European Union and European Economic Area (EU/EEA) was higher than the combined burden of 31 other infectious diseases under surveillance by ECDC [1,2]. The estimated number of healthcare-associated infections (HAI) used in the study was based on the data of the first ECDC point prevalence survey (PPS) of HAI and antimicrobial use in acute care hospitals (ACH) from 2011 to 2012 [3] and did not take into account HAI occurring in other healthcare facilities. In particular, ECDC had previously estimated that the number of residents with an HAI on any given day in European long-term care facilities (LTCF) was of the same order of magnitude as the number of patients with an HAI on any given day in ACH [4-6].

In the period from 2016 to 2017, ECDC organised two PPS of HAI and antimicrobial use: the second PPS in ACH and the third PPS in LTCF in the EU/EEA. The objective of the current study was to report on the HAI and antimicrobial resistance results of both surveys and to estimate the combined total number of HAI on any given day and the number of HAI per year from 2016 to 2017 in the EU/EEA.

## Methods

### Participation of countries

All EU/EEA countries and EU candidate and potential candidate countries were invited to organise a national PPS in ACH and LCTF in their country in any of four periods (April to June or September to November of 2016 or 2017). For reasons of feasibility at national level, the PPS in ACH and LCTF could be organised during different periods. Data were collected according to two specific standardised ECDC protocols [7,8]. All countries used the ECDC protocols and included all HAI types except for one country (Norway) for ACH and four countries (France, the Netherlands, Norway and Sweden) for LCTF. Norway used national protocols with the same case definitions as in the ECDC protocols, but provided fewer details and did not require the inclusion of all types of HAI. LTCF data from France and the Netherlands were also collected using national protocols not including all types of HAI. LTCF protocols in France, the Netherlands and Norway all included urinary tract infections, lower respiratory tract infections and skin infections, in addition other HAI types varying by country. Surveys in separate healthcare administrations in the United Kingdom (UK), i.e. England, Northern Ireland, Scotland and Wales, were organised independently and results were reported separately.

### Selection of participating facilities and patients

It was recommended that countries selected the participating ACH and LCTF by systematic random sampling from national lists ranked by type and size to ensure optimal country representativeness. For each country, the required sample size was calculated for an estimated prevalence of 6% for ACH and 4% for LCTF, based on the results of the previous PPS [3,6], with an absolute precision of 1%. Representativeness was categorised as optimal, good, poor or very poor, depending on the sampling method of the facilities, the number of included patients/residents and the number of included facilities [7,8]. For example, ‘optimal representativeness’ meant that the country performed systematic sampling of at least 25 healthcare facilities or included at least 75% of all facilities or beds at national level, and achieved the recommended sample size.

For ACH, the protocol recommended that data from a single ward should be collected on one single day and that the time frame for data collection for all wards of a single hospital would not exceed 3 weeks. For LCTF, it was recommended to collect data on a single day, except for larger LCTF.

We included all patients/residents present on the hospital ward or LTCF at 8:00 on the day of the PPS and still present at the time of day when the PPS was performed. In addition, LTCF residents needed to be full-time residents (i.e. living 24 hours a day in the LTCF). Patients/residents who were temporarily absent from their room, e.g. for diagnostic procedures, had to be included.

### Case definitions

Case definitions for HAI differed for ACH and for LCTF, reflecting differences in access to diagnostic methods between the two settings, as well as the specific signs and symptoms of infection in elderly LTCF residents [7,8]. For both PPS, an HAI was defined as active on the day of the PPS when signs and symptoms were present on the date of the PPS, or when signs and symptoms were no longer present but the patient/resident was still receiving treatment for that infection on the date of the PPS. HAI present on admission were included in both protocols. In the LTCF protocol, HAI associated with a stay in any other healthcare facility – another LTCF or a hospital –­ were included. In the ACH protocol, however, only HAI imported from other ACH were included, excluding HAI present on admission associated with a previous LTCF stay. LTCF data in France and Sweden did not include HAI imported from other healthcare facilities.

### Data analysis

Data were analysed with Stata, version 14.1 (StataCorp, Texas, United States). The prevalence of HAI was expressed as the percentage of patients/residents with at least one HAI on the day of the PPS. To account for clustering within ACH or LCTF, 95% confidence intervals (CI) were calculated using the svy proportion command in Stata. Overall weighted prevalence percentages were calculated by applying the country-specific prevalence on the number of occupied beds in each country and summing up the total number of patients with at least one HAI for EU/EEA countries. National denominator data were obtained by questionnaire from national survey coordinators, from Eurostat data if national denominator data were not submitted [9-11] or from the previous PPS if Eurostat data were missing or incomplete [3,4,6]. To estimate the total number of HAI or patients with at least one HAI for the whole EU/EEA, the average results from participating EU/EEA countries were applied to the national denominator data from non-participating EU/EEA countries. For data collected using national protocols which did not include all types of HAI, imputation of non-included types of HAI was done based on EU/EEA averages to make prevalence percentages comparable. In ACH, imputation resulted in adding 7.3% (36/495) of patients with HAI in Norway. In LCTF, imputation resulted in adding 5.8% (12/206) of residents with HAI in France, 6.9% (11/160) in the Netherlands and 7.6% (9/119) in Norway, or 0.8% (32/3,780) overall. As these imputations were done for the aggregated national results, correction of CI for clustering within LCTF could not be applied for these countries and binomial exact CI were used instead.

### Antimicrobial resistance

Antimicrobial resistance (AMR) in HAI was evaluated using two indicators: a composite index of AMR and the percentage of carbapenem-resistant Enterobacteriaceae. The composite index of AMR was calculated as the percentage of resistant isolates for the ‘first level’ AMR markers in the PPS protocols divided by the sum of the isolates for which results from antimicrobial susceptibility testing (AST) were reported. These first level markers were *Staphylococcus aureus* resistant to meticillin (MRSA), *Enterococcus faecium* and *Enterococcus faecalis* resistant to vancomycin, Enterobacteriaceae resistant to third-generation cephalosporins, and *Pseudomonas aeruginosa* and *Acinetobacter baumannii* resistant to carbapenems. The percentage of resistant isolates was not calculated when less than 10 isolates with known AST results were reported. The composite index of AMR at country level was validated by examining the correlation with the composite AMR index calculated from EARS-Net data from 2016, including all components of the index except AST results for Enterobacteriaceae other than *Escherichia coli* and *Klebsiella pneumoniae* because they are not included in EARS-Net [12,13]. Correlations were analysed using the Spearman correlation coefficient rho and the R-squared (R^2^) and regression coefficient from linear regression.

### Prevalence to incidence conversion

Estimates of the total number of HAI and patients acquiring at least one HAI per year in ACH were based on prevalence to incidence conversion using the Rhame and Sudderth formula [14]. Details of the method are reported in the ECDC PPS report for 2011 and 2012 [3]. In addition, sensitivity analyses of the conversion were carried out using a method developed by Willrich et al. (personal communication: Niklas Willrich, 24 May 2018), in which the estimates of the length of stay were based on a Grenander estimator for discrete monotonously decreasing distributions [15].

In LCTF, only the number of HAI could be estimated. As LTCF usually are permanent residences, HAI do not prolong the length of stay of a resident as they do in ACH. Therefore, the incidence of HAI in LCTF per year was estimated by multiplying the prevalence by 365 days and dividing it by the duration of infection (in days), with a correction for an average occupancy of LTCF beds of 95%, calculated from institutional denominator data. The duration of infection was estimated, by type of HAI, from the date of onset to the date of the PPS, using the median duration of HAI until the day of the PPS multiplied by 2.

### Validation studies

It was strongly recommended that all participating EU/EEA countries perform validation studies of their national PPSs. For the PPS in ACH, ECDC also offered financial support to national institutions coordinating PPS so that they could organise validation studies with a minimum requirement to re-examine 250 patient charts in five ACH. For both the PPS in ACH and that in LCTF, the objective was to estimate representative validity parameters at the EU/EEA level rather than at country level ([16]; ACH validation protocol available from the authors on request). Validation studies were performed by national validation teams composed of members of the national coordination teams, using the ECDC HAI case definitions as gold standard. Validation results were calculated for each country, by matching patients included in the validation sample with their corresponding data collected in the primary PPS. The percentage of false positives (FP) and false negatives (FN) was calculated from the matched analysis and applied to the total national database to calculate the sensitivity and specificity for each country, as several countries selected high prevalence wards for validation to improve precision as recommended by the validation study protocol. For correction of the EU/EEA prevalence of HAI, the EU/EEA mean FN and FP were applied to the total number of patients. The validation-corrected HAI prevalence was converted using the Rhame and Sudderth formula to estimate the corrected HAI incidence and total number of patients in ACH with at least one HAI per year in the period 2016 to 2017.

To calculate CI around EU/EEA estimates, the number of patients with at least one HAI obtained from the lower and upper limits of the country-specific 95% CIs were summed up and divided by the total number of occupied beds (for prevalence) or the total number of discharges (for estimated incidence) in the EU/EEA. These ‘cumulative 95% CI’ (95% cCI) therefore reflect a larger, more conservative uncertainty than would be obtained by calculating 95% CI on the EU/EEA totals, which is in accordance with the limitations of the prevalence measurement and the uncertainty inherent to the conversion of prevalence to incidence.

## Results

### Point prevalence survey in acute care hospitals

#### Participation

In total, 1,735 hospitals from 28 EU/EEA countries and one EU candidate country (Serbia) participated in the second PPS of HAI and antimicrobial use in European ACH in the period 2016 to 2017. Counting UK administrations separately, the country representativeness of the sample was optimal in 20 countries, good in 10, and poor in two countries. After adjustment for over-representation of countries contributing more than 20,000 patients to the PPS, 325,737 patients from 1,275 ACH remained in the final sample. Aggregated results were only reported for the EU/EEA, corresponding to 310,755 patients from 1,209 ACH. The distribution of the type of ACH and the percentage of patients requiring intensive care by country is shown in Table 1.

**Table 1:** Key characteristics of healthcare facilities, patients and residents included in the point prevalence survey (PPS) samples, PPS in acute care hospitals (n = 1,275) and long-term care facilities (n = 2,242), 30 EU/EEA countries, Serbia and the former Yugoslav Republic of Macedonia, 2016–2017

Country	ACH								LTCF								
	Number of hospitals		Type of ACH					Intensive care patients (%)	Number of LCTF		Type of LTCF				Residents in (a) + (b) + (c)		
	Country total	In PPS sample	Primary	Secondary	Tertiary	Specialised	Unknown		Country total	In PPS sample	General nursing home (a)	Residential home (b)	Mixed LTCF (c)	Other LTCF types	>85 years-old (%)	Urinary catheter (%)	Recent surgery (%) (past 30 days)
Austria	162	49	25	11	2	11	0	4.0	817	14	0	7	5	2	35.8	10.8	1.0
Belgium	197	43	27	9	7	0	0	4.9	1,559	86	79	0	0	7	56.5	3.1	0.9
Bulgaria	241	12	1	4	7	0	0	6.9	33	NP	NA	NA	NA	NA	NA	NA	NA
Croatia	32	34	6	15	9	4	0	6.0	325	8	0	0	8	0	40.9	3.1	1.1
Cyprus	83	8	2	4	2	0	0	9.6	90	13	7	0	4	2	54.8	8.0	4.8
Czech Republic	144	45	2	30	11	2	0	8.1	73	11	0	4	5	2	NA	NA	NA
Denmark	52	NP	NA	NA	NA	NA	NA	NA	827	95	0	0	95	0	51.8	9.0	1.7
Estonia	27	23	10	7	1	4	1	3.3	59	NP	NA	NA	NA	NA	NA	NA	NA
Finland	59	51	18	16	14	2	1	3.8	1,928	157	148	0	1	8	51.4	4.2	0.6
France	1,237	50	32	10	6	2	0	3.8	9,744	91	91	0	0	0	61.6	1.6	0.8
Germany	1,857	49	25	7	4	13	0	5.0	10,389	84	55	15	12	2	49.6	8.6	1.3
Greece	123	42	1	23	16	2	0	7.6	263	13	0	0	13	0	48.8	12.1	0.7
Hungary	94	38	14	10	6	7	1	2.8	1,177	111	65	9	1	36	25.3	1.9	0.7
Iceland	8	2	0	1	1	0	0	5.2	43	NP	NA	NA	NA	NA	NA	NA	NA
Ireland	60	60	9	17	7	27	0	3.0	578	185	75	0	34	76	47.7	7.0	1.5
Italy	1,134	56	13	14	25	4	0	6.0	3219	215	61	85	50	19	54.0	12.1	1.3
Latvia	24	14	0	9	3	2	0	3.5	82	NP	NA	NA	NA	NA	NA	NA	NA
Lithuania	64	62	25	26	8	3	0	2.8	154	26	0	0	26	0	12.4	0.8	0.3
Luxembourg	12	12	2	5	1	3	1	5.9	62	16	15	1	0	0	58.4	5.3	1.5
Malta	4	4	1	1	1	1	0	4.8	41	11	0	8	3	0	51.1	5.0	0.6
The Netherlands	79	19	10	8	1	0	0	6.0	700	57	0	0	57	0	43.0	6.6	3.5
Norway	53	43	11	9	4	0	19	6.3	907	62	62	0	0	0	NA	10.0	3.4
Poland	936	80	22	20	23	15	0	3.8	373	25	12	12	0	1	30.5	19.4	0.9
Portugal	225	93	24	40	18	9	2	4.2	360	268	0	0	132	136	29.6	15.1	0.9
Romania	311	40	16	10	3	11	0	6.4	628	NP	NA	NA	NA	NA	NA	NA	NA
Slovakia	107	50	20	11	7	12	0	5.2	677	69	27	0	32	10	28.3	3.1	1.1
Slovenia	21	20	0	11	3	6	0	5.8	90	NP	NA	NA	NA	NA	NA	NA	NA
Spain	576	96	17	39	32	5	3	5.0	5,387	46	0	0	46	0	48.1	5.1	5.1
Sweden	144	NP	NA	NA	NA	NA	NA	NA	2,300	417	285	0	0	132	57.9	9.9	2.1
UK–England	158	32	0	19	10	3	0	3.4	17,473	NP	NA	NA	NA	NA	NA	NA	NA
UK–Northern Ireland	16	16	6	4	2	4	0	3.2	445	70	0	15	55	0	44.8	5.0	0.6
UK–Scotland	46	45	12	14	7	12	0	2.8	873	52	34	17	1	0	43.9	8.5	0.3
UK–Wales	21	21	6	10	4	1	0	3.7	795	30	9	7	12	2	49.7	7.8	1.7
EU/EEA	8,307	1,209	357	414	245	165	28	4.6%	62,471	2,232	1025	180	592	435	45.6%	6.7%	1.5%
EU/EEA (n, %, mean of countries)	252	100%	29.5%	34.2%	20.3%	13.6%	2.3%	4.9%	1,893	100%	45.9%	12.3%	22.3%	19.5%	44.8%	7.3%	1.5%
Former Yugoslav Republic of Macedonia	ND	NP	NA	NA	NA	NA	NA	NA	21	4	3	0	1	0	15.3	8.8	0.7
Serbia	66	66	1	45	14	6	0	6.5	90	6	0	0	6	0	28.1	6.1	0.6

ACH: acute care hospital; EU/EEA: European Union/European Economic Area; LTCF: long-term care facility; NA: not applicable; ND: no data collected in national protocol; NP: did not participate; PPS: point prevalence survey; UK: United Kingdom.

Country data representativeness was poor in Bulgaria and the Netherlands for the PPS in ACH and poor in Austria, Croatia, Cyprus, Greece, Luxembourg, Malta and Poland for the PPS in LTCF. The Czech Republic only submitted data on institutional indicators.

#### Prevalence and estimated incidence of healthcare-associated infections

A total of 19,626 HAI were reported in 18,287 patients with HAI (1.07 HAI per infected patient). The prevalence of patients with at least one HAI in the EU/EEA sample was 5.9% (country range: 2.9–10.0%; Table 2). The prevalence varied between 4.4% (2,177/49,381 patients) in primary care hospitals (n = 333) to 7.1% (7,591/104,562 patients) in tertiary care hospitals (n = 222) and was highest in patients admitted to intensive care units, where 19.2% (2,751/14,258) patients had at least one HAI compared with 5.2% (15,536/296,397) on average for all other specialties combined (Supplement).

**Table 2:** Prevalence and estimated incidence of healthcare-associated infections in European acute care hospitals, 28 EU/EEA countries and Serbia, 2016–2017 (n = 325,737 patients)

Country	Patients in PPS sample	Patients with at least one HAI in PPS sample (HAI prevalence)^a^			Validation-corrected HAI prevalence^b^	Occupied beds in the country (average per day)	Patients with at least one HAI on a given day, estimated		Hospital discharges annually in the country	HAI incidence, estimated		Patients with at least one HAI, annually, estimated	
	n	n	%	95% CI	%	n	n	95% CI	n	%	95% CI	n	95% CI
Austria	13,461	541	4.0	3.4–4.7	NR	36,351	1,461	1,243–1,716	2,707,753	2.3	1.5–3.3	62,306	40,978–89,762
Belgium	11,800	856	7.3	6.4–8.3	NR	37,651	2,731	2,397–3,109	1,858,726	5.4	3.7–7.6	101,110	68,186–141,713
Bulgaria ^c^	2,200	76	3.5	1.7–6.8	NR	25,324	875	434–1,733	1,632,089	1.8	0.9–3.8	29,572	13,909–61,597
Croatia	10,466	551	5.3	4.5–6.2	NR	11,047	581	495–683	667,849	4.1	2.8–5.6	27,129	18,937–37,561
Cyprus	1,036	85	8.2	5.4–12.4	ND	1,437	118	77–178	166,295	4.8	2.5–8.7	8,010	4,158–14,541
Czech Republic	15,117	1,015	6.7	5.9–7.6	NR	40,691	2,732	2,413–3,090	2,260,239	5.4	3.9–7.3	122,313	87,039–165,208
Estonia	4,220	178	4.2	2.4–7.3	NR	4,582	193	111–332	222,363	3.3	1.6–6.6	7,393	3,558–14,761
Finland	9,079	803	8.8	7.5–10.4	NR	15,894	1,406	1,187–1,660	915,892	5.1	3.3–7.5	46,735	30,053–68,350
France	16,522	965	5.8	4.9–7.0	NR	159,810	9,334	7,823–11,116	11,330, 996	4.1	2.7–5.9	467,961	311,830–671,498
Germany	11,324	409	3.6	2.8–4.7	NR	400,132	14,452	11,087–18,789	19,480,504	3.1	1.9–4.8	604,495	373,766–938,383
Greece	9,401	938	10.0	8.5–11.6	NR	18,252	1,821	1,559–2,121	1,562,761	4.3	3.1–5.7	66,487	48,386–89,068
Hungary	20,588	818	4.0	3.3–4.8	NR	46,134	1,833	1,516–2,212	2,226,485	3.5	2.1–5.4	78,095	46,906–120,082
Iceland	633	40	6.3	0.8–36.8	5.7	642	41	5–237	39,198	6.7	0.6–48.6	2,609	239–19,038
Ireland	10,333	633	6.1	5.0–7.5	NR	10,932	670	546–820	705,000	4.2	2.7–6.3	29,671	18,846–44,323
Italy	14,773	1,186	8.0	6.8–9.5	NR	167,619	13,457	11,362–15,899	8,930,979	6.0	4.2–8.3	534,709	373,705–740,544
Latvia	3,807	140	3.7	2.6–5.2	4.9	5,127	189	132–268	300,575	2.5	1.4–4.1	7,447	4,322–12,399
Lithuania	12,415	359	2.9	2.1–4.0	3.2	14,613	423	301–590	705,224	2.6	1.3–4.6	18,046	9,322–32,167
Luxembourg	2,018	103	5.1	4.0–6.5	8.5	1,860	95	75–120	74,782	3.4	2.1–5.3	2,569	1,560–3,995
Malta	961	60	6.2	5.2–7.4	7.9	972	61	51–72	72,909	2.6	1.9–3.4	1,877	1,380–2,507
The Netherlands^c^	4,441	170	3.8	3.4–4.3	NR	24,167	925	826–1,036	1,700,000	2.3	1.6–3.2	39,585	27,525–54,115
Norway^d^	9,628	495	5.1	4.1–6.4	ND	10,505	540	430–677	776,203	2.4	1.5–3.6	18,767	11,873–28,340
Poland	21,712	1,249	5.8	4.8–6.9	4.7	120,492	6,931	5,764–8,317	8,254,611	3.5	2.3–5.0	289,602	193,881–415,274
Portugal	16,982	1,544	9.1	8.1–10.2	7.8	27,907	2,537	2,236–2,841	1,128,245	5.9	4.4–7.8	66,860	49,568–87,500
Romania	11,443	417	3.6	2.8–4.7	5.9	57,091	2,080	1,610–2,682	3,674,275	2.6	1.7–4.0	97,257	62,340–146,893
Slovakia	9,145	370	4.1	3.1–5.3	NR	20,279	820	630–1,066	1,005,003	3.1	2.1–4.6	31,519	20,848–46,607
Slovenia	5,720	373	6.5	5.8–7.3	ND	5,581	363	322–409	380,077	4.4	3.3–5.6	16,635	12,630–21,441
Spain	19,546	1,516	7.8	7.1–8.5	NR	84,908	6,586	5,983–7,243	5,247,215	4.9	3.6–6.4	255,169	186,398–335,644
UK–England	20,148	1,297	6.4	5.4–7.6	NR	96,774	6,230	5,264–7,358	9,450,142	2.2	1.4–3.2	205,722	130,191–303,990
UK–Northern Ireland	3,813	234	6.1	4.8–7.9	5.8	4,965	305	236–392	302,008	3.5	1.8–5.9	10,527	5,559–17,841
UK–Scotland	11,623	504	4.3	3.5–5.3	NR	11,448	496	406–606	1,156,473	2.2	1.5–3.2	25,539	16,992–36,977
UK–Wales	6,400	362	5.7	4.7–6.7	6.0	6,715	380	318–453	827,634	2.2	1.3–3.3	17,880	10,595–27,545
**Participating EU/EEA countries^a,e^**	**310,755**	**18,287**	**5.5**	**4.5–6.7**	**6.5**	**1,469,903**	**80,665**	**66,864–97,824**	**89,762,505**	**3.7**	**2.4–5.3**	**3,293,595**	**2,185,484–4,789,661**
Serbia	14,982	650	4.3	3.5–5.4	NR	18,920	821	656–1,024	988,383	3.3	2.3–4.6	32,337	22,714–45
EU/EEA, corrected**^e,f^**	NA	NA	5.5	4.5–6.7	6.5	1,503,881	82,713	67,674–99,256	91,885,503	3.7	2.4–5.3	3,372,146	2,220,554–4,854,535
**EU/EEA, corrected after validation**	NA	NA	**6.5**	**5.4–7.8**	NA	1,503,881	**98,166**	**81,022–117,484**	91,885,503	**4.1**	**3.4–4.9**	**3,758,014**	**3,122,024–4,509,617**

CI: confidence interval; EU/EEA: European Union/European Economic Area; HAI: healthcare-associated infection; NA: not applicable; ND: validation study not done NR: validation study not representative of country PPS sample; PPS: point prevalence survey; UK: United Kingdom.

^a^ Country-weighted HAI prevalence for the EU/EEA = estimated number of patients with at least one HAI a single day / occupied beds.

^b^ Validation-corrected prevalence of patients with at least one HAI: only given for countries that reached national representativeness for their national validation study (at least 75% of recommended sample size of 750 validated patients and/or validation of at least 75% of included hospitals).

^c^ Poor country representativeness in Bulgaria and the Netherlands.

^d^ Norway used a national PPS protocol requiring imputation of non-included types of HAI for 24 hospitals.

^e^ Cumulative 95% CI for the EU/EEA. Cumulative sums are rounded and may differ from the sum of the individual rounded country estimates.

^f^ Corrected for non-participating EU countries with estimation for Denmark and Sweden combined.

When extrapolated to the average daily number of occupied beds per country, the weighted HAI prevalence was 5.5% (95% cCI: 4.5–6.6%). The weighted annual incidence of patients acquiring at least one HAI per year in the period 2016 to 2017, estimated using prevalence to incidence conversion, was 3.7 (95% cCI: 2.4–5.3) patients per 100 admissions. National PPS validation studies were carried out by 28 countries (UK administrations counted separately) in a total of 236 ACH in the EU/EEA. National validation teams re-examined 12,228 patient charts independently from the primary PPS surveyors. These studies showed that on average, 2.3% (country range: 0.3–5.6%) of patients who were reported as not having a HAI actually had an HAI (false negatives) while one in five (mean: 20.3%, country range: 0–46.2%) patients reported as having an HAI did not have an HAI (false positives), resulting in a mean sensitivity of HAI detection of 69.4% (country range: 40.1–94.4%) and a mean specificity of 98.8% (country range: 96.1–100%). When correcting for these results, the adjusted prevalence of patients with at least one HAI was estimated at 6.5% (95% cCI: 5.4–7.8%). Using the Rhame and Sudderth formula to convert the latter percentage, the corrected annual incidence was estimated at 4.1 (95% cCI: 3.4–4.9) patients per 100 admissions. Applying the EU/EEA averages to denominator data from non-participating EU/EEA countries (Denmark and Sweden), this resulted in an estimated total of 98,166 (95% cCI: 81,022–117,484) patients with at least one HAI on any given day and 3,758,014 (95% cCI: 3,122,024–4,509,617) patients with at least one HAI per year in the period 2016 to 2017 in ACH in the EU/EEA.

#### Types of HAI and isolated microorganisms

The most frequently reported types of HAI were respiratory tract infections (21.4% pneumonia and 4.3% other lower respiratory tract infections), urinary tract infections (18.9%), surgical site infections (18.4%), bloodstream infections (10.8%) and gastro-intestinal infections (8.9%), with *C. difficile* infections accounting for 44.6% of the latter or 4.9% of all HAI. Twenty-three per cent of HAI were present on admission. One third of HAI on admission were surgical site infections. Country-weighted prevalence percentages and estimated numbers of HAI per year are shown in Table 3. After correction for non-participating countries and validation, a total of 4.5 million (95% cCI: 2.6–7.6 million) HAI were estimated to occur per year in the period 2016 to 2017 in ACH in the EU/EEA.

**Table 3:** Country-weighted prevalence and estimated incidence of healthcare-associated infections (HAI) by type of HAI in European acute care hospitals (n = 19,626) and long-term care facilities (n = 3,858), 30 EU/EEA countries, 2016–2017

Type of HAI	Acute care hospitals								Long-term care facilities							
	HAI in PPS sample		Country-weighted HAI prevalence		Estimated HAI on a given day, EU/EEA^a^		Estimated annual HAI, EU/EEA^a^		HAI in PPS sample		Country-weighted HAI prevalence		Estimated HAI on a given day, EU/EEA^a^		Estimated annual HAI, EU/EEA^a^	
	N	% total	n	95% cCI	N	95% cCI	n	95% cCI	n	% total	%	95% cCI	n	95% cCI	n	95% cCI
Respiratory tract infection																
Pneumonia	4,200	21.4	1.26	0.96–1.68	18,935	14,398–25,265	862,084	567,728–1 283,203	143	3.7	0.15	0.06–0.32	4,948	1,946–10 658	112,868	44,390–243,134
Other lower respiratory tract infection^b^	838	4.3	0.24	0.15–0.41	3,568	2,208–6,192	183,232	91,731–376,990	847	22.0	0.88	0.59–1.14	29,010	19,412–37,826	1,058,853	708,542–1 380,653
Common cold/influenza	NI	NA	NA	NA	NA	NA	NA	NA	290	7.5	0.29	0.13–0.51	9,678	4,368–16,782	441,543	199,312–765,693
Urinary tract infection	3,710	18.9	1.10	0.85–1.43	16,491	12,822–21,455	869,941	572,105–1,278,951	1,233	32.0	1.29	0.87–1.66	42,687	28,898–54,825	1,298,388	878,983–1,667,596
Surgical site infection	3,601	18.3	1.08	0.81–1.44	16,130	12,185–21,715	518,182	293,036–858,222	66	1.7	0.09	0.03–0.20	2,829	944–6,500	57,366	19,133–131,803
Bloodstream infection	2,116	10.8	0.69	0.48–1.00	10,294	7,241–15,097	375,050	227,552–613,624	19	0.5	0.04	0.01–0.07	1,168	193–2,389	23,692	3,908–48,442
Gastrointestinal infection																
*Clostridium difficile* infection	951	4.8	0.32	0.21–0.51	4,786	3,105–7,721	189,526	105,154–340,978	37	1.0	0.05	0.01–0.14	1,787	424–4,755	18,118	4,296–48,206
Other gastrointestinal infection	792	4.0	0.24	0.14–0.41)	3,549	2,108–6,166	144,926	64,880–312,212	75	1.9	0.1	0.03–0.20	3,187	1,012–6,473	145,409	46,184–295,333
Skin and soft tissue infection	823	4.2	0.21	0.13–0.36	3,146	1,900–5,451	108,269	45,149–242,816	828	21.5	0.83	0.51–1.19	27,459	17,021–39,307	626,415	388,293–896,687
Eye, ear, nose or mouth infection	557	2.8	0.16	0.09–0.35	2,400	1,278–5 194	123,091	54,155–303,206	183	4.7	0.17	0.08–0.31	5,712	2,707–10,369	173,733	82,323–315,390
Systemic infection	1,069	5.4	0.29	0.17–0.52	4,388	2,586–7,799	251,237	110,732–549,877	35	0.9	0.04	0.01–0.08	1,223	286–2,534	37,201	8,691–77,061
Other infection	969	4.9	0.30	0.19–0.50	4,518	2,867–7,574	154,138	65,647–332,357	102	2.6	0.12	0.04–0.24	3,878	1,366–8,077	117,958	41,556–245,683
All types of HAI, EU/EEA^a^	19,626	100	NA	NA	88,204	62,697–129,630	3,779,677	2,197,869–6,492,437	3,858	100	NA	NA	133,565	78,576–200,494	4,111,544	2,425,610–6,115,682
**All types of HAI, EU/EEA, corrected after validation**	NA	NA	NA	NA	**104,177**	**74,743–152,575**	**4,464,159**	**2,620,139–7,641,606**	NA	NA	NA	NA	143,565	64,736–260,655	**4,422,629**	**1,998,384–7,950,784**

cCI: cumulative 95% confidence interval (sum of country-specific lower respectively upper country interval limits); EU/EEA: European Union/European Economic Area; HAI: healthcare-associated infection; NA: not applicable; NI: not included in protocol; PPS: point prevalence survey.

^a^ After correction for non-participating countries. Cumulative sums are rounded and may differ from the sum of the individual rounded country estimates.

^b^ Other lower respiratory tract infections included bronchitis, tracheobronchitis, bronchiolitis, tracheitis, lung abcess or empyema, without evidence of pneumonia.

A total of 13,085 microorganisms were reported in 10,340 (52.7%) HAI. The 10 most frequently isolated microorganisms were *E. coli* (16.1%), *S. aureus* (11.6%), *Klebsiella* spp. (10.4%), *Enterococcus* spp. (9.7%), *P. aeruginosa* (8.0%), *C. difficile* (7.3%), coagulase-negative staphylococci (7.1%), *Candida* spp. (5.2%), *Enterobacter* spp. (4.4%) and *Proteus* spp. (3.8%).

#### Antimicrobial resistance in healthcare-associated infections and correlation with EARS-Net data

AST data were available for 8,031 (88.9%) of 9,034 microorganisms included in the composite index of AMR. The index was 31.6% overall (mean of countries: 30.8%) and varied from 0% in Iceland to 68.9% in Romania. The index by country was strongly correlated with the index calculated from 2016 EARS-Net data on invasive isolates (Spearman’s correlation coefficient *rho*: 0.93; p < 0.001; R^2^: 0.86. Figure) and was on average 36% higher for HAI in ACH from the PPS than in the EARS-Net data (mean of countries in EARS-Net: 20.3%). Carbapenem resistance in Enterobacteriaceae was 6.2% overall (mean of countries: 5.9%) and ranged from 0% in Estonia, Finland, Iceland, Lithuania and UK–Northern Ireland to 43.7% in Greece (Table 4). This indicator also correlated well with carbapenem resistance in *E. coli* and *K. pneumoniae* in EARS-Net data (Spearman’s *rho*: 0.76; p < 0.001) and was on average 45% higher in HAI in ACH from the PPS than in EARS-Net data (mean of countries in EARS-Net: 2.6%). The total number of patients acquiring an HAI with at least one resistant microorganism was estimated at 291,067 (95% cCI: 162,417–504,270) patients for the composite index of AMR and 31,696 (95% cCI: 14,611–78,205) patients for carbapenem-resistant Enterobacteriaceae.

**Figure:**
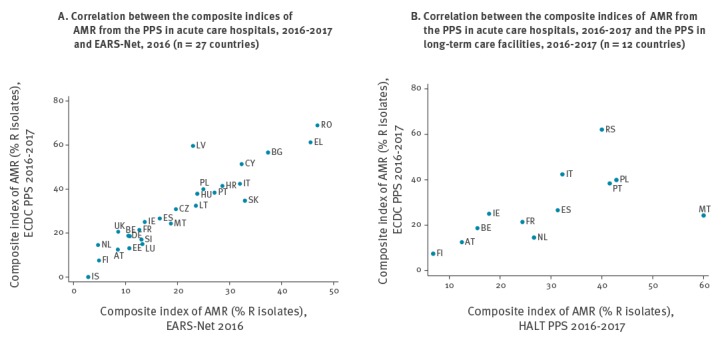
Correlations of composite index of antimicrobial resistance, EU/EEA countries and Serbia, 2016–2017

ACH: acute care hospital; AMR: antimicrobial resistance; AT: Austria; BE: Belgium; BG: Bulgaria; CY: Cyprus; CZ: Czech Republic; DE: Germany; EARS-Net: European Antimicrobial Resistance Surveillance Network; ECDC: European Centre for Disease Prevention and Control; EE: Estonia; EL: Greece; ES: Spain; FI: Finland; FR: France; HALT: Healthcare-associated infections in LTCF project; HR: Croatia; HU: Hungary; IE: Ireland; IS: Iceland; IT: Italy; LT: Lithuania; LCTF: long-term care facility; LU: Luxembourg; LV: Latvia; MT: Malta; NL: the Netherlands; NO: Norway; PL: Poland; PPS: point prevalence survey; PT: Portugal; RO: Romania; RS: Serbia; SI: Slovenia; SK: Slovakia; UK: United Kingdom.

Composite index of AMR: *Staphylococcus aureus* resistant to meticillin, *Enterococcus faecium* and *Enterococcus faecalis* resistant to vancomycin, Enterobacteriaceae resistant to third-generation cephalosporins, *Pseudomonas aeruginosa* and *Acinetobacter baumannii* resistant to carbapenems; EARS-Net: Enterobacteriaceae other than *Escherichia coli* and *Klebsiella pneumoniae* not included. Other species represented 32.5% of tested Enterobacteriaceae in ACH. France: percentage non-susceptible (resistant + intermediate) isolates instead of percentage resistant isolates. In addition to poor representativeness of participating LCTF in Malta, specimens in these LCTF were known to be taken predominantly in cases of treatment failure (panel B).

**Table 4:** Composite index of antimicrobial resistance in bacteria from healthcare-associated infections in acute care hospitals (n = 8,413) and long-term care facilities (n = 565), 30 EU/EEA countries, Serbia and the former Yugoslav Republic of Macedonia^a^, 2016–2017

Country	Acute care hospitals^a^								Long-term care facilities^a^			
	Composite index of AMR				Carbapenem-resistant Enterobacteriaceae				Composite index of AMR		Carbapenem-resistant Enterobacteriaceae	
	Tested isolates	Resistant isolates	Estimated annual HAI		Tested isolates	Resistant isolates	Estimated annual HAI		Tested isolates	Resistant isolates	Tested isolates	Resistant isolates
	n	%	n	95% CI	n	%	n	95% CI	n	%	n	%
Austria^b^	217	12.4	1,759	713–3,984	124	0.8	55	8–387	16	12.5	12	0.0
Belgium	495	18.6	8,458	4,422–14,621	318	1.3	261	104–654	45	15.6	34	0.0
Bulgaria^b^	53	56.6	8,687	3,189–23,328	30	10.0	2,014	479–8,291	NP	NA	NA	NA
Croatia^b^	280	41.4	3,823	2,491–5,808	114	5.3	300	80–1,053	6	NA	4	NA
Cyprus^a,b^	37	51.4	1,070	431–2,380	15	6.7	19	3–119	0	NA	NA	NA
Czech Republic^a^	627	30.8	16,348	9,726–25,665	393	0.8	87	30–261	NP^a^	NA	NA	NA
Denmark^a^	NP	NA	UNK	NA	NA	NA	UNK	NA	0	NA	0	NA
Estonia	107	13.1	462	138–1,398	58	0.0	0	NA	NP	NA	NA	NA
Finland	188	7.4	298	139–619	92	0.0	0	NA	44	6.8	36	0.0
France^a^	738	21.4	44,953	21,316–86,180	413	0.5	785	129–4,943	41	24.4	35	14.3
Germany	197	18.8	27,228	13,378–52,651	95	2.1	1,769	420–7,444	2	NA	1	NA
Greece^b^	456	61.2	10,605	7,809–14,193	197	43.7	4,157	2,467–6,831	2	NA	1	NA
Hungary	256	37.9	5,383	2,578–9,837	126	0.8	41	6–289	7	NA	6	NA
Iceland	15	0.0	0	NA	10	0.0	0	NA	NP	NA	NA	NA
Ireland	192	25.0	1,206	454–2,704	107	0.9	45	6–306	28	17.9	12	8.3
Italy	555	42.3	63,930	39,969–98,909	306	16.7	11,660	6,489–20,554	93	32.3	67	5.6
Latvia	47	59.6	804	309–2,043	19	5.3	38	4–356	NP	NA	NA	NA
Lithuania	108	32.4	1,509	680–3,224	35	0.0	0	NA	2	.	3	NA
Luxembourg^b^	67	14.9	79	26–228	38	2.6	4	0–46	3	.	2	NA
Malta^b^	33	24.2	195	69–544	25	4.0	23	0–2,216	15	60.0	7	NA
The Netherlands^b^	110	14.5	2,755	1,201–6,052	73	2.7	167	40–688	15	26.7	13	0.0
Norway^a^	ND	NA	UNK	NA	ND	NA	UNK	NA	ND	NA	ND	NA
Poland^b^	531	39.9	30,356	18,445–47,719	262	6.9	2,535	976–6,569	21	42.9	13	0.0
Portugal	829	38.4	9,177	5,431–14,287	462	6.9	1,062	347–2,643	65	41.5	47	10.6
Romania	164	68.9	13,913	7,377–25,458	80	33.8	3,475	1,726–6,923	NP	NA	NA	NA
Slovakia	164	34.8	3,061	1,543–5,848	101	2.0	247	60–1,022	8	NA	4	NA
Slovenia	194	17.0	969	397–2,087	117	1.0	3	1–17	NP	NA	NA	NA
Spain	926	26.6	25,722	15,842–38,973	512	4.1	2,632	1,136–5,609	134	31.3	82	0.0
Sweden	NP	NA	UNK	NA	NA	NA	UNK	NA	3	NA	1	NA
UK–England	370	20.5	7,634	3,950–13,560	205	1.5	316	101–986	NP	NA	NA	NA
UK–Northern Ireland	40	25.0	333	145–758	17	0.0	0	NA	2	NA	0	NA
UK–Scotland^a^	ND	NA	UNK	NA	ND	NA	UNK	NA	ND	NA	ND	NA
UK–Wales	35	37.1	351	67–1,213	8	NA	0	NA	1	NA	0	NA
**EU/EEA^c^**	**8,031**	**31.6**	**291,067**	**162,417–504,270**	**4,352**	**6.2**	**31,696**	**14,611–78,205**	**553**	**28.0**	**380**	**4.2**
Former Yugoslav Republic of Macedonia	NP	NA	UNK	NA	ND	NA	UNK	NA	2	NA	1	NA
Serbia	382	62.0	7,555	4,516–12,230	201	25.4	1,435	801–2,481	10	40.0	8	NA

AMR: antimicrobial resistance; CI: confidence interval; EU/EEA: European Union/European Economic Area; HAI: healthcare-associated infection; NA: not applicable; ND: no data collected in national PPS; NP: did not participate; PPS: point prevalence survey; UNK: unknown; UK: United Kingdom.

^a^Antimicrobial resistance data were not reported by Norway and UK–Scotland in the PPS in acute care hospitals and by Denmark, Norway and UK–Scotland in the PPS in long-term care facilities. Cyprus did not submit case-based HAI data for long-term care facilities. The Czech Republic only collected institutional indicators for the PPS in long-term care facilities. For France, the percentage of non-susceptible (resistant + intermediate) isolates is given instead of the percentage resistant isolates.

^b^Country data representativeness was poor in Bulgaria and the Netherlands for the PPS in acute care hospitals and poor in Austria, Croatia, Cyprus, Greece, Luxembourg, Malta and Poland for the PPS in long-term care facilities.

^c^Cumulative 95% confidence intervals for the EU/EEA. Cumulative sums are rounded and may differ from the sum of the individual rounded country estimates.

Composite index of AMR: *Staphylococcus aureus* resistant to meticillin, *Enterococcus faecium* and *Enterococcus faecalis* resistant to vancomycin, Enterobacteriaceae resistant to third-generation cephalosporins, *Pseudomonas aeruginosa* and *Acinetobacter baumannii* resistant to carbapenems. Enterobacteriaceae selected for the AMR markers: *Escherichia coli, Klebsiella* spp., *Enterobacter* spp., *Proteus* spp., *Citrobacter* spp., *Serratia* spp. and *Morganella* spp. The percentage of resistance was not calculated if less than 10 isolates were reported.

### Point prevalence survey in long-term care facilities

#### Participation

In total, 3,062 LCTF from 24 EU/EEA countries and two EU candidate countries (Serbia and the former Yugoslav Republic of Macedonia) participated in the third PPS of HAI and antimicrobial use in European LCTF in the period 2016 to 2017. Counting UK administrations separately, good or optimal representativeness of the national sample was obtained in 18 of 24 EU/EEA countries. After adjustment for over-representation, 117,138 residents from 2,221 LCTF were included for analysis. The main aggregated results were reported for 80.5% of participating LCTF, i.e. general nursing homes (n = 1,025), residential homes (n = 176) and mixed LCTF (n = 587), corresponding to 102,301 residents and 1,788 LCTF in EU/EEA countries. The characteristics of LCTF and residents by country are shown in Table 1.

#### Prevalence of healthcare-associated infections

A total of 3,858 HAI were reported in 3,780 residents with HAI (1.02 HAI per infected resident). The prevalence of residents with at least one HAI was 3.7% (country range: 0.9–8.5%). When extrapolated to the average number of occupied LTCF beds per country, the weighted HAI prevalence in LCTF was 3.6% (95% cCI: 2.9–4.5%). Validation of the PPS in LCTF was performed for 953 residents in 17 LCTF in 10 countries. National validation teams found 1.1% (95% CI: 0.5–2.0%) false-negative residents and 19.6% (95% CI: 9.4–33.9%) false-positive residents, yielding a sensitivity of 73.7% and a specificity of 99.2% when applied on the total EU/EEA database. The country-weighted, validation-corrected HAI prevalence was 3.9% (95% cCI: 2.4–6.0%). Applying the EU/EEA prevalence to denominator data from non-participating EU/EEA countries, the total number of residents with at least one HAI on any given day in EU/EEA LCTF was estimated at 129,940 (95% cCI: 79,570–197,625) residents (Table 5).

**Table 5:** Prevalence of healthcare-associated infections in long-term care facilities, 23 EU/EEA countries^a^, Serbia and the former Yugoslav Republic of Macedonia, 2016–2017 (n = 103,763 residents)

Country	LCTF included in analysis	Residents included in analysis	Residents with at least one HAI in PPS sample (HAI prevalence)^b^			HAI from other facility^c^	HAI prevalence origin own LTCF^d^	LTCF beds in the country	Residents with at least one HAI on a given day, estimated	
	n	n	n	%	95%CI	%	n	n	n	(95% CI)
Austria^e^	12	2,065	105	5.1	2.8–8.9	6.5	4.6	72,602	3,504	1,966–6,145
Belgium	79	8,206	354	4.3	3.6–5.1	4.9	3.6	146,462	5,997	5,037–7,152
Croatia^e^	8	1,607	15	0.9	0.4–1.9	13.3	0.7	37,249	329	159–679
Cyprus^e^	11	312	15	4.8	2.7–7.8	ND	ND	3,436	157	89–255
Denmark	95	3,346	175	5.2	4.5–6.1	5.0	4.8	42,668	2,120	1,808–2,481
Finland	149	5,914	208	3.5	3.0–4.1	5.1	3.2	50,373	1,685	1,436–1,967
France^f^	91	6,957	206	3.0	2.6–3.4	ND	3.0	687,936	19,352	16,831–22,134
Germany	82	6,705	115	1.7	1.3–2.3	13.0	1.3	852,849	13,936	10,209–18,878
Greece^e^	13	812	51	6.3	3.7–10.5	3.8	5.9	10,849	647	381–1,079
Hungary	75	7,670	73	1.0	0.7–1.4	4.1	0.9	57,929	523	369–743
Ireland	109	5,613	276	4.9	4.2–5.8	6.0	4.5	30,531	1,427	1,207–1,682
Italy	196	11,417	442	3.9	3.3–4.6	13.6	3.1	186,872	6,870	5,787–8,149
Lithuania	26	3,438	32	0.9	0.4–1.9	15.6	0.6	11,722	104	50–212
Luxembourg^e^	16	1,616	30	1.9	1.1–3.0	0.0	1.8	6,966	123	75–199
Malta^e^	11	2,485	76	3.1	1.6–5.9	12.3	2.3	5,035	146	75–281
The Netherlands^f^	57	4,547	160	3.5	3.0–4.1	5.0	3.2	92,000	3,075	2,624–3,580
Norway^f^	62	2,447	119	4.9	4.0–5.8	2.5	4.6	39,583	1,829	1,521–2,178
Poland^e^	24	2,281	90	3.9	2.1–7.3	7.6	3.5	17,291	649	345–1,198
Portugal	132	3,633	214	5.9	4.5–7.6	15.9	4.3	8,400	470	362–608
Slovakia	59	5,091	108	2.1	1.5–3.0	4.5	2.0	27,497	554	392–778
Spain	46	6,808	579	8.5	7.0–10.3	18.9	6.2	372,306	30,064	24,688–36,501
Sweden	285	3,604	57	1.6	1.2–2.1	ND	1.6	93,000	1,396	1,051–1,864
UK–Northern Ireland	70	2,614	97	3.7	2.9–4.7	7.1	3.4	15,924	561	443–710
UK–Scotland	52	2,147	125	5.8	4.5–7.5	2.4	5.3	37,746	2,087	1,610–2,697
UK–Wales	28	966	58	6.0	4.4–8.2	0.0	6.0	24,646	1,405	1,026–1,915
**Participating EU/EEA countries^b,g^**	**1,788**	**102,301**	**3,780**	**3.6**	**2.9–4.5**	**8.9**	**3.1**	**2,931,872**	**99,008**	**79,539–124,064**
Former Yugoslav Republic of Macedonia	4	294	10	3.4	2.3–4.9	0.0	2.7	1,166	38	26–55
Serbia	6	1,168	37	3.2	1.9–5.1	7.3	2.8	19,654	592	362–960

EU/EEA, corrected^g,h^	NA	NA	NA	3.6	2.9–4.5	NA	NA	3,486,999	117,754	94,599–147,553
**EU/EEA, corrected after validation**	NA	NA	NA	**3.9**	**2.4–6.0**	NA	NA	3,486,999	**129,940**	**79,570–197,625**

EU/EEA: European Union/European Economic Area; HAI: healthcare-associated infection; LTCF: long-term care facility; PPS: point prevalence survey; ND: no data collected in national protocol; UK: United Kingdom.

^a^ The Czech Republic only submitted data on institutional indicators from 11 LCTF and was not included in the current analysis.

^b^ Country-weighted HAI prevalence for the EU/EEA = estimated number of residents with at least one HAI on a single day / occupied beds; occupied beds = number of LTCF beds × average occupancy of 0.95.

^c^ Percentage of HAI imported from a hospital or another LTCF; not included in France and Sweden, and unknown for Cyprus (aggregated data).

^d^ HAI prevalence for HAI with the own LTCF as origin, i.e. excluding HAI imported from other healthcare facilities and HAI with unknown origin (Supplement).

^e^ Country data representativeness was poor in Austria, Croatia, Cyprus, Greece, Luxembourg, Malta and Poland.

^f^ France, the Netherlands and Norway used a national protocol which required imputation of non-included types of HAI.

^g^ Cumulative 95% confidence intervals for the EU/EEA.

^h^ Corrected for non-participating EU/EAA countries with estimation for Bulgaria, Czech Republic, Estonia, Iceland, Latvia, Romania, Slovenia and UK–England combined.

#### Types of healthcare-associated infections and isolated microorganisms

The most frequently reported types of HAI in LCTF were respiratory tract infections (33.2% overall, 3.7% pneumonia, 22.0% other lower respiratory tract infections, 7.2% common cold/pharyngitis, 0.3% influenza), urinary tract infections (32.0%) and skin infections (21.5%). The majority of the reported HAI (84.7%) were associated with the LTCF where the PPS was performed, while 7.5% and 1.4% were associated with a hospital or another LTCF, respectively. The origin was unknown for 6.4% of HAI in LCTF. Country-weighted prevalence percentages and estimated number of infections per year are given by type of HAI in Table 3. The total number of HAI in LCTF in the EU/EEA, after applying EU averages for non-participating EU/EEA countries and correcting for validation, was estimated at 4.4 million (95% cCI: 2.0–8.0 million). Microbiological data in LCTF were available for 742 (19.2%) HAI. The 10 most frequently isolated bacteria were *E. coli* (30.7%), *S. aureus* (12.3%), *Klebsiella* spp. (11.4%), *Proteus* spp. (10.6%), *P. aeruginosa* (7.1%), *Enterococcus* spp. (4.8%), *C. difficile* (4.4%), *Streptococcus* spp. (2.8%) *Enterobacter* spp. (2.1%) and coagulase-negative staphylococci (1.9%).

#### Antimicrobial resistance in healthcare-associated infections and correlation with data from the hospital point prevalence survey

AST results were available for 553 (77.6%) of 713 microorganisms included in the composite index of AMR. The index could be calculated for 11 countries with at least 10 isolates, and was 28.0% overall, ranging from 6.8% in Finland to 60.0% in Malta (Table 4). The composite index of AMR correlated well between ACH and LCTF, although Malta was an outlier (Figure, Spearman’s *rho* excluding Malta: 0.86; p < 0.001; R^2^ = 0.69). On average, the percentage of resistant microorganisms was similar in both settings (regression coefficient excluding Malta: 1.08). Carbapenem resistance in Enterobacteriaceae in LCTF was 4.2% overall and did not correlate significantly with the percentage in ACH (Table 4).

## Discussion

Because both the PPS in ACH and that in LCTF were performed during 2016 and 2017, this provided the first opportunity to estimate the prevalence, incidence and annual number of HAI for ACH and for LCTF in the EU/EEA for the same time period. As expected, the overall prevalence of HAI was higher in ACH than in LCTF, also after correction based on validation study results. However, when estimating the total number of HAI, both settings were shown to have similarly high numbers of HAI annually. In total, 8.9 million distinct HAI episodes were estimated to occur annually in ACH and LCTF in the EU/EEA. In ACH, where the incidence per patient could be calculated, the number of patients with at least one HAI was estimated at 3.8 (95% cCI: 3.1–4.6) million patients per year in the period 2016 to 2017. 

The country-weighted HAI prevalence before validation correction in ACH of 5.5% (95% cCI: 4.5–6.7%) was similar to the HAI prevalence of 5.7% (95% cCI: 4.5–7.4%) in the ECDC PPS in ACH in the period 2011 to 2012 [3]. The unweighted HAI prevalence in LCTF of 3.7% before correction was only slightly higher than the prevalence of 3.4% found in the ECDC PPS in LCTF in 2013 [6], although imported HAI were included in the period 2016 to 2017. The final corrected country-weighted HAI prevalence estimates of 6.5% in ACH and 3.9% in LCTF were higher because they were corrected for the results of the validation studies, which made the current estimates more robust than the previous estimates. Similarly, the estimated incidence and number of HAI in ACH presented in this study were higher than the number estimated in the ECDC PPS from 2011 to 2012 [3] because of the correction for the results of the validation study and should therefore not be interpreted as an increase for ACH compared with the period 2011 to 2012.

The strong correlation of the composite indices of AMR in the ECDC PPS in ACH with the EARS-Net data supports the validity of AMR data collected in the PPSs. The 36% higher percentage of resistant isolates in HAI in the ECDC PPS was expected given that EARS-Net only includes data from invasive isolates, i.e. from bloodstream infections and meningitides, and that a large proportion of isolates reported to EARS-Net are from community-associated bloodstream infections, especially for MRSA and *E. coli* resistant to third-generation cephalosporins. However, the fact that the composite index of AMR in LCTF was at the same level as in ACH, at least in countries where both indicators could be calculated, is of concern. Even though the low testing frequency in LCTF is probably biased towards HAI which are non-responsive to empiric treatment, this finding emphasises the urgent need to reinforce measures to improve infection prevention and control, antimicrobial stewardship as well as microbiological laboratory support for LCTF.

Our study has several limitations. Firstly, the small number of countries and LCTF that performed validation studies in the PPS in LCTF resulted in less robust prevalence estimates for LCTF than for ACH, even though the LTCF validation results could be used at the EU/EEA level. Secondly, the conversion from prevalence to incidence using the Rhame and Sudderth formula has been shown to have several limitations in itself, especially for smaller samples [17,18]. The estimates depend on the estimators used, as not all data can be acquired from a cross-sectional prevalence study. Nevertheless, sensitivity analyses that we performed with more recent estimator methodology (personal communication: Niklas Willrich, 24 May 2018) [15] yielded EU/EEA estimates which were close to those reported here, with few exceptions at individual country level. Especially considering the wide CI, this gave more weight to our estimates (Supplement). Thirdly, the estimates also strongly depended on the quality of the national denominator data of the number of beds, and, for ACH, discharges and patient days. Providing reliable national denominator data has been shown to be difficult for many countries that sometimes provided estimates rather than precise numbers, especially for LCTF. In addition, as national denominator data for specialised LCTF were only available in two countries, a specific incidence for these types of LTCF could not be estimated. In several countries, however, the number of beds for these LCTF are included in the total number of LTCF beds for the country. We only reported results for the main types of LTCF, as these types were consistently included in all countries. Fourthly, the number of residents with at least one HAI each year could not be estimated for LCTF in the EU/EEA. Longitudinal HAI incidence data would be required to produce such estimates. Fifthly, three countries preferred using their national PPS protocols for LCTF and one country for ACH, resulting in less robust estimates. Sixthly, the total number of HAI with resistant pathogens could only be estimated for ACH because of the poor availability of microbiological results in LCTF. Moreover, the annual incidence estimates of HAI with resistant pathogens in ACH are underestimated because: (i) in almost half of the HAI in ACH, a microorganism was not reported, (ii) for 11% of the reported microorganisms, AST results were not yet available on the day of the PPS and (iii) correction for countries without data and correction for validation was not performed. Despite these limitations, the estimated number of HAI with carbapenem-resistant Enterobacteriaceae using Rhame and Sudderth conversion in our study (31,696 infections, of which 27,393 were HAI with carbapenem-resistant *E. coli* or *K. pneumoniae*) was close to the number of 33,172 infections with carbapenem-resistant *E. coli* or *K. pneumoniae* recently estimated by Cassini et al. using a totally different methodology [19].

The main strengths of this study are its large sample size and the use of standardised protocols for data collection and validation across participating ACH and LCTF. Despite some countries providing less representative samples, these PPSs as a whole offer a representative picture of HAI in the EU/EEA, with benchmarks to help direct future action in ACH and LCTF in participating countries.

## Conclusion 

This study reports, to our knowledge, the most accurate and robust estimates of the total number of HAI in healthcare facilities in the EU/EEA to date, and confirms that HAI, and AMR in bacteria responsible for HAI, represent a significant healthcare issue and public health challenge for the EU/EEA. Considering that previous studies have shown that HAI in ACH alone are responsible for more deaths in the EU/EEA than all other infectious diseases under surveillance at European level [1,2], and that our study showed that there are as many HAI in LTCF as there are in ACH, more focus needs to be dedicated to the prevention of HAI and AMR, through the application of available recommendations and guidelines [20-25], in both ACH and LTCF. 

## Notes

**Members of the Healthcare-Associated Infections Prevalence Study Group:** Elisabeth Presterl (Medical University Vienna, Vienna, Austria; Elisabeth.presterl@meduniwien.ac.at) Reinhild Strauss (Federal Ministry for Labour, Social Security, Health and Consumer Protection (BMAGSK) Vienna, Austria;reinhild.strauss@sozialministerium.at) Eline Vandael (Sciensano, Brussels, Belgium; Eline.Vandael@sciensano.be) Boudewijn Catry (Sciensano, Brussels, Belgium; Boudewijn.Catry@sciensano.be) Elina Dobreva (National Center of Infectious and Parasitic Diseases (NCIPD), Sofia, Bulgaria; elina_g@abv.bg) Nadezhda Vladimirova (National Center of Infectious and Parasitic Diseases (NCIPD), Sofia, Bulgaria; nvladimirova@ncipd.org) Zrinka Bošnjak (University of Zagreb School of Medicine, University Hospital Centre Zagreb, Zagreb, Croatia; zbosnjak@kbc-zagreb.hr) Ana Budimir (University of Zagreb School of Medicine, University Hospital Centre Zagreb, Zagreb, Croatia; abudimir@kbc-zagreb.hr) Elena Gabriel (Ministry of Health, Nicosia, Cyprus; EGabriel@ns.moh.gov.cy) Linos Hadjihannas (Ministry of Health, Nicosia, Cyprus; lhadjihannas@mphs.moh.gov.cy) Vlastimil Jindrák (National Institute of Public Health, Prague, Czech Republic; vlastimil.jindrak@gmail.com) Dana Hedlová (National Institute of Public Health, Prague, Czech Republic; dana.hedlova@uvn.cz) Christian Stab Jensen (Statens Serum Institut (SSI), Copenhagen, Denmark; csj@ssi.dk) Pille Märtin (West Tallinn Central Hospital, Health Board), Tallinn, Estonia; pille.martin@keskhaigla.ee) Piret Mitt (Tartu University Hospital, Tartu, Estonia; piret.mitt@kliinikum.ee) Emmi Sarvikivi (National Institute for Health and Welfare, Helsinki, Finland; emmi.sarvikivi@thl.fi) Dinah Arifulla (National Institute for Health and Welfare, Helsinki, Finland; dinah.arifulla@thl.fi) Saija Toura (National Institute for Health and Welfare, Helsinki, Finland; saija.toura@thl.fi) Anne Berger-Carbonne (National Public Health Agency (Santé publique France), Saint Maurice, France; anne.berger-carbonne@santepubliquefrance.fr) Anne Savey (CPias Auvergne-Rhône-Alpes, University Hospital (HCL), Lyon, France; anne.savey@chu-lyon.fr) Côme Daniau (National Public Health Agency (Santé publique France), Saint Maurice, France; come.daniau@santepubliquefrance.fr) Claudia Ruscher (Robert Koch Institute, Berlin, Germany; RuscherC@rki.de) Petra Gastmeier (Institute of Hygiene and Environmental Medicine, Charité – University Medicine Berlin, Berlin, Germany) Seven Aghdassi (Institute of Hygiene and Environmental Medicine, Charité – University Medicine Berlin, Berlin, Germany) Nicole Schmidt (Robert Koch-Institute, Berlin, Germany; schmidtn@rki.de) Achilleas Gikas (Department of Internal Medicine, University Hospital of Heraklion, Heraklion, Crete, Greece; gikas.achilles@uoc.gr) Meropi Gkika (Faculty of Medicine, University of Crete, Heraklion, Greece; gikameri@yahoo.gr) Symeon H. Panagiotakis (University Hospital of Heraklion, Heraklion, Crete, Greece; simeongpan@hotmail.com) Andrea Kurcz (Ministry of Human Capacities, Budapest, Hungary; andrea.kurcz@emmi.gov.hu) Ágnes Hajdu (Ministry of Human Capacities, Budapest, Hungary; agnes.hajdu1@emmi.gov.hu) István Veress (Ministry of Human Capacities, Budapest, Hungary; istvan.veress@emmi.gov.hu) Ólafur Guðlaugsson (Landspitali University Hospital, Reykjavik, Iceland; olafgudl@landspitali.is) Karen Burns (Health Protection Surveillance Centre, Beaumont Hospital & Royal College of Surgeons in Ireland, Dublin, Ireland; karen.burns1@hse.ie) Helen Murphy (Health Protection Surveillance Centre, Dublin, Ireland; helen.murphy@hpsc.ie) Carla M. Zotti (Department of Public Health and Pediatric Sciences, University of Turin, Turin, Italy; carla.zotti@unito.it) Francesca Quattrocolo (Department of Public Health and Pediatric Sciences, University of Turin, Turin, Italy; francesca.quattrocolo@unito.it) Angelo D'Ambrosio (Department of Public Health and Pediatric Sciences, University of Turin, Turin, Italy; angelo.dambrosio@unito.it) Elina Dimiņa (Centre for Disease Prevention and Control, Riga, Latvia; elina.dimina@spkc.gov.lv) Aija Vilde (Pauls Stradins Clinical University Hospital, Riga, Latvia; aija.vilde@stradini.lv) Ieva Kisielienė (Institute of Hygiene, Vilnius, Lithuania; ieva.kisieliene@rvul.lt) Rolanda Valintėlienė (Institute of Hygiene, Vilnius, Lithuania; rolanda.valinteliene@hi.lt) Martine Debacker (Ministère de la santé - Direction de la santé, Luxembourg, Luxembourg; martine.debacker@ms.etat.lu) Murielle Weydert (Ministère de la Famille, de l’Intégration et à la Grande Région, Luxembourg, Luxembourg; murielle.weydert@fm.etat.lu) Branka Petrovska Basovska (National Institute of Public Health, Skopje, former Yugoslav Republic of Macedonia; branka.petrovska@yahoo.com) Dragan Kochinski (National Institute of Public Health, Skopje, former Yugoslav Republic of Macedonia; dragan.kocinski@gmail.com) Michael A Borg (Mater Dei Hospital & University of Malta, Msida, Malta; michael.a.borg@gov.mt) Elizabeth Scicluna (Mater Dei Hospital, Msida, Malta; elizabeth.a.scicluna@gov.mt) Mark Bonanno (St Vincent De Paul Long Term Care Facility, Luqa, Malta; mark.bonanno@gov.mt) Titia EM Hopmans (National Institute for Public Health and the Environment (RIVM), Bilthoven, The Netherlands; titia.hopmans@rivm.nl) Mayke BG Koek (National Institute for Public Health and the Environment (RIVM), Bilthoven, The Netherlands; mayke.koek@rivm.nl) Linda Verhoef (National Institute for Public Health and the Environment (RIVM), Bilthoven, The Netherlands; linda.verhoef@rivm.nl ; current address l.p.b.verhoef@nvwa.nl) Kati Halonen (National Institute for Public Health and the Environment (RIVM), Bilthoven, The Netherlands; kati.halonen@rivm.nl) Hanne-M. Eriksen (Norwegian Institute of Public Health, Oslo, Norway; hmer@fhi.no) Horst Bentele (Norwegian Institute of Public Health, Oslo, Norway; hobe@fhi.no) Nina Sorknes (Norwegian Institute of Public Health, Oslo, Norway; Nina.Sorknes@fhi.no) Anna Różańska (Chair of Microbiology, Faculty of Medicine Jagiellonian University Medical School, Krakow, Poland; a.rozanska@uj.edu.pl) Jadwiga Wojkowska-Mach (Chair of Microbiology, Faculty of Medicine Jagiellonian University Medical School, Krakow, Poland; jadwiga.wojkowska-mach@uj.ed.pl) Isabel Neves, (Direção-Geral da Saúde (DGS), Lisbon, Portugal; Isabel.Neves@ulsm.min-saude.pt) Margarida Valente, (Direção-Geral da Saúde (DGS), Lisbon, Portugal; margaridavalente@dgs.min-saude.pt) Pedro Pacheco (Direção-Geral da Saúde (DGS), Lisbon, Portugal; pedrojorgepacheco@gmail.com) Roxana Ioana Serban (National Institute of Public Health (NIPH), Bucharest, Romania; roxana.serban@insp.gov.ro) Andreea Sorina Niculcea (National Institute of Public Health (NIPH), Bucharest, Romania; andreea.niculcea@insp.gov.ro) Ljiljana Markovic-Denic (University of Belgrade, Faculty of Medicine, Belgrade, Serbia; markovic.denic@gmail.com) Ivana Cirkovic (University of Belgrade, Faculty of Medicine, Belgrade, Serbia; cirkoviciv@yahoo.com) Mária Štefkovičová (Alexander Dubcek University in Trenčín and Regional Public Health Authority in Trenčín, Slovakia; tn.stefkovicova@uvzsr.sk) Slavka Litvová (Regional Public Health Authority in Trenčín, Slovakia; tn.litvova@uvzsr.sk) Irena Klavs (National Institute of Public Health, Ljubljana, Slovenia; Irena.Klavs@nijz.si) Mojca Serdt (National Institute of Public Health, Ljubljana, Slovenia; Mojca.Serdt@nijz.si) Angel Asensio (Hospital Universitario Puerta de Hierro-Majadahonda, Madrid, Spain; aasensio.hpth@salud.madrid.org) Mireia Cantero (Hospital Universitario Puerta de Hierro-Majadahonda, Madrid, Spain; mireia.cantero@salud.madrid.org) Pilar Gallego Berciano (National Centre for Epidemiology, Instituto de Salud Carlos III, Madrid, Spain. Biomedical Research Centre Network for Epidemiology and Public Health (CIBERESP); pgallego@isciii.es) Jenny Hellman (The Public Health Agency of Sweden, Solna, Sweden; jenny.hellman@folkhalsomyndigheten.se) Tomas Söderblom (The Public Health Agency of Sweden, Solna Sweden; tomas.soderblom@folkhalsomyndigheten.se) Alan Johnson (Public Health England, London, UK; Alan.Johnson@phe.gov.uk) Muhammad Sartaj (HSC Public health Agency, Belfast, Northern Ireland, UK; muhammad.sartaj@hscni.net) Mark McConaghy (Public Health Agency, Belfast, Northern Ireland, UK mark.mcconaghy@hscni.net) Shona Cairns (Health Protection Scotland National Services Scotland, Glasgow, UK; shona.cairns@nhs.net) Wendy Harrison (Public Health Wales, Cardiff, Wales, UK; wendy.harrison2@wales.nhs.uk) Liselotte Diaz Högberg (European Centre for Disease Prevention and Control; Liselotte.Diaz-Hogberg@ecdc.europa.eu) Alessandro Cassini (European Centre for Disease Prevention and Control; Alessandro.Cassini@ecdc.europa.eu) Olivia Aya Nakitanda (European Centre for Disease Prevention and Control; Olivia.Aya.Nakitanda@ecdc.europa.eu) Ole Heuer (European Centre for Disease Prevention and Control; Ole.Heuer@ecdc.europa.eu)
