# Erratum for Euro Surveill. 2018;23(46)

**DOI:** 10.2807/1560-7917.ES.2018.23.47.181122e1

**Published:** 2018-11-22

**Authors:** Eurosurveillance editorial team

**Affiliations:** 1 European Centre for Disease Prevention and Control (ECDC), Stockholm, Sweden

In the article ‘Prevalence of healthcare-associated infections, estimated incidence and composite antimicrobial resistance index in acute care hospitals and long-term care facilities: results from two European point prevalence surveys, 2016 to 2017’, published on 15 November 2018, the list of members of the Healthcare-Associated Infections Prevalence Study Group was left out in the original publication and added on Friday, 16 November 2018.

